# Different expression of Micro RNA-126, 133a and 145 in aorta and saphenous vein samples of patients undergoing coronary artery bypass graft surgery

**DOI:** 10.15171/jcvtr.2019.07

**Published:** 2019-02-16

**Authors:** Ahmadreza Jodati, Seyed Mohammadbagher Pirouzpanah, Nazila Fathi Maroufi, Masoud Pezeshkian, Naser Safaie, Hossain Bijanpour, Amir Mahdi Khamaneh, Ali Mota, Mohammad Nouri

**Affiliations:** ^1^Cardiovascular Research Center, Tabriz University of Medical Sciences, Tabriz, Iran; ^2^Stem cell and Regenerative Medicine Institute, Tabriz University of Medical Sciences, Tabriz, Iran; ^3^Lvts u1148 Inserm Institut Galilee Universite Paris 13, Paris, France; ^4^Department of Biochemistry & Clinical Laboratory, Faculty of Medicine, Tabriz University of Medical Sciences, Tabriz, Iran; ^5^Student Research Committee, Tabriz University of Medical Sciences, Tabriz, Iran; ^6^Department of Molecular Medicine, Faculty of Advanced Medical Sciences, Tabriz University of Medical Sciences, Tabriz, Iran

**Keywords:** MicroRNA-126, 133a and 145, Coronary Artery Disease, Aorta, Saphenous Vein

## Abstract

***Introduction:*** microRNAs (miRNAs) are highly conserved, noncoding RNA molecules that regulate gene expression on the post-transcriptional level. Some evidence indicates that microRNAs dysfunction plays a crucial role in human disease development. The role of microRNAs in cardiac growth, hypertrophy, heart failure, cardiovascular complications in diabetes and many other hearth conditions are demonstrated. In this study we aimed to evaluate the expression of six microRNAs (mir-100, mir-126, mir-127, mir-133a, mir-133b and mir-145) that have been shown to overexpress in aortic and carotid plaques.

***Methods:*** Thirty Coronary Artery Disease patients who underwent elective coronary artery bypass graft surgery were enrolled in the study. The expression patterns of six miRNAs (mir-100, mir-126, mir-127, mir-133a, mir-133b, and mir-145) were examined in 30 patients of whom we obtained aorta and saphenous vein samples.

***Results:*** In three miRNAs, mir-100, mir-127 and mir-133b, we did not obtain expression data from real-time experiments. We found that the expression level of mir-126, mir-133a and mir145 were lower in aorta in comparison with saphenous vein. Mir-126 was highly expressed in saphenous vein samples (13.8±1.1) when compared with aorta samples (20.2±1.1), although mir133a was highly expressed in saphenous vein samples (16.1±0.5) when compared with the aorta (17.9±1.5). Expression of mir-145 saphenous vein samples was also dramatically higher than aorta (7.2±0.5 versus 10.8±0.6) that was statistically significant (*P*<0.05).

***Conclusion:*** Understanding the role of miRNAs in cardiovascular physiology and diseases might suggest miRNA- based therapeutic methods in the management of coronary artery disease.

## Introduction

Heart failure remains the important trouble to present humanity and treating physician with the most important reason for hospital entrance in adults more than the age of 65. In accordance with World Health Organization (WHO), major factors contributing heart failure are coronary artery disease (CAD), hypertension, diabetes mellitus; atrial fibrillation and valvular heart disease are the five.^1^ Micro-RNAs (miRNAs) are highly conserved, non-coding RNA molecules, about 22 nucleotides long,^2^ that regulate gene expression on the post-transcriptional level.^3,4^ It is estimated that about one-third of the genes are regulated by miRNAs and a single miRNA can regulate as many as 200 mRNAs.^5^ miRNAs act as endogenous repressors of target genes, either by inhibiting translation and/or by promoting degradation of the miRNA, or alternatively by increasing translation.^6^ miRNAs have been known to participate in control of most developmental and biological processes ranging from cell cycle control, cell growth and differentiation, apoptosis and so on.^7^

Evidence indicates that miRNAs dysfunction play crucial roles in human disease development.^7^ The role of miRNAs in cardiac growth, hypertrophy, heart failure, cardiovascular complications in diabetes and many other hearth conditions are demonstrated.^8^ It is also showed that miRNAs dysfunction may have a critical association with cardiac dysfunction. Different expression of miRNAs in several conditions such as hypertrophy, arrhythmias, atrial fibrillation, heart failure has been studied.^9,10^ Up-regulation of some miRNAs in atherosclerotic plaques have also been shown.^11,12^ The role of miRNAs in vascular smooth muscle cell proliferation, differentiation, and in atherosclerosis has been demonstrated.^13,14^ High levels of miR-1 and miR-133a were shown to be present in acute myocardial infarction (AMI), without increase of serum cardiac troponin or serum creatinine phosphokinase (CPK). As a result, it can be used as a marker of cardiomyocyte damage or death.^15^ Cardiac-specific miRNAs can be used for the recognition of AMI have been describing by a variety of researchers. Cardiac specific miRNAs-1/133/208b/499 may have the potential for future diagnostic markers for AMI.^15,16^ Most studies that have been carried out on their relationship with heart disease have been performed in plasma or serum samples. There are very few studies that have been done on vascular plaques or vascular tissues. On the other hand, all the components involved in heart disease have not yet been fully identified and require further research. Given the discussions in this study we aimed to evaluate the expression of six miRNAs (mir-100, mir-126, mir-127, mir-133a, mir-133b and mir-145) that have been shown to over-expressed in aortic and carotid plaques.^17,18^ For this reason the expression of these micro-RNAs in aorta and saphenous vein samples obtained during coronary artery bypass surgery and the expression of these miRNAs are assessed.

## Materials and Methods

### Patients and samples

Thirty CAD patients who underwent elective coronary artery bypass graft (CABG) surgery enrolled in the study. The study was approved by the local ethics committee of Tabriz University of Medical Sciences. All the patients were clinically symptomatic at presentation and proved to have coronary artery stenosis in angiographic examination. Tiny samples of aorta and saphenous vein (1 to 1.5 g) were taken during the bypass surgery. Samples were rinsed with PBS and were frozen in liquid nitrogen and stored in liquid nitrogen container until RNA extraction.

For all patients, biochemical and laboratory profiles were analyzed in the central library, Shahid Madani Heart Center, using a Hitachi 912 analyzer (Japan). Total serum cholesterol and high density lipoprotein (HDL) cholesterol were measured by an enzymatic colorimetric method, and HDL cholesterol was measured after precipitation with phosphor-tungstate and magnesium ions. Triacylglyceroles were also quantified with an enzymatic colorimetric method. Low-density lipoprotein (LDL) cholesterol was calculated using the Friedewald formula.^19^

### RNA extraction and cDNA synthesis

The samples were transferred into RNase free tube and ground. The total RNA was extracted from the samples using miRCURY RNA isolation kit (EXIQON) which intended to extract small RNAs, according to the manufacturer’s instruction. The total RNA concentration was measured using NanoDrop 1000 Spectrophotometer (thermo). RNA samples subjected to DNase I digestion (Thermo Scientific, USA) before cDNA synthesis. One hundred nanograms of total RNA for each sample was reverse-transcribed using miRCURY LNA Universal RT microRNA PCR kit (EXIQON) according to the manufacturer’s instruction. For relative quantification of miRNAs (mir-100, mir-126, mir-127, mir133a, mir-133b, mir-145), PCR amplification kit (miRCURY LNA Universal RT microRNA PCR, EXIQON) with specific primers provided in the kit was used. miR-191 was used as reference gene for data normalization.^20^

### Statistical analysis

Statistical analysis was done with SPSS, version 17 (SPSS Inc., USA). Mean values and standard deviations are presented for descriptive. To compare mean values of miRNAs in aorta and saphenous vein samples, paired sample *t* test was used. To compare mean values of miRNAs in relation to patients BMI independent sample *t* test was used. Exact *P* value of <0.05 with 95% confidence interval was considered as statistically significant.

## Results

### Patient’s characteristics

Demographic characteristics and laboratory findings for the study group are summarized in Table 1. The majority of patients who underwent CABG surgery were elderly, and male patients are predominant. Based on WHO, BMI classification 14 patients were overweight (BMI, 25.00-29.99) and 11 of them were obese (BMI ≥ 30.00).

**Table 1:** Demographic characteristics and laboratory findings

**Variable**	
Age (y)	60.7±9.6
Gender (Male/Female)	23/7
BMI (kg/m^2^)	28.1±3.08
BMI>30 (n)	11
Total cholesterol (mg/dL)	186±54
LDL cholesterol (mg/dL)	121±44
HDL cholesterol (mg/dL)	38±10
Triglycerides(mg/dL)	156±82
Fasting blood glucose (mg/dL)	114±22
Na (mg/dL)	141.37±0.22
K (mg/dL)	4.38±0.26
Diabetes mellitus type 2	4/30(13.33%)
Hypertension	18/30(60%)
Hyperlipidemia	13/30(43.33%)
Aortic stenosis	26/30(86.66%)
Number of diseased vessels	
One-vessel	7/30(23.33%)
Two-vessel	10/30(33.3%)
Three-vessel	13/30(43.33%)

### miRNA expression pattern

The expression patterns of six miRNAs (mir-100, mir-126, mir-127, mir-133a, mir-133b, and mir-145) were examined in 30 patients with CAD from whom we obtained aorta and saphenous vein (Table 2). In three miRNAs, mir-100, mir-127 and mir-133b, we did not obtain expression data from real-time experiments. We found that the expression level of mir-126, mir-133a and mir-145 were lower in aorta in comparison with saphenous vein. These lower expression levels were showed with higher Ct values in real-time quantitative PCR experiments. Mir-126 were essentially highly expressed in saphenous vein samples (13.8±1.1) when compared with aorta samples (20.2±1.1) that were statistically significant (*P* < 0.05, 95% CI 1.35 to 2.25). We showed that mir-133a were highly expressed in saphenous vein samples (16.1±0.5) when compared with aorta (17.9±1.5) (*P* < 0.05, 95% CI 5.4 to 7.6). As showed in Table 2, the expression of mir-145 saphenous vein samples was also dramatically higher than aorta (7.2±0.5 versus 10.8±0.6) (*P* < 0.05, 95% CI 3.3 to 3.8). Fold changes of each miR expression were calculated using the 2-DDCt method. The comparative cycle threshold (∆∆Ct) was defined as a difference between ∆Ct (aneurysmal/ normalization) minus ∆Ct (nonaneurysmal/normalization).

**Table 2:** The expression patterns of six miRNAs (mir-100, mir-126, mir-127, mir-133a, mir-133b, and mir-145)

**miRNA**	**Aorta**	**Saphenous**	***P*** value*
mir-100	ND	ND	-
mir-126	20.2±1.1**	13.8±1.1**	0.000
mir-127	ND	ND	-
mir-133a	17.9±1.5**	16.1±0.5**	0.002
mir-133b	ND	ND	-
mir-145	10.8±0.6**	7.2±0.5**	0.000

ND: Not determined. *Values are obtained by paired sample *t* test. ** Values are express as mean ± SD.

When the expression of miRNAs in aorta and saphenous vein analyzed considering BMIs of the patients, the results are as followed (Table 3). In this context of patient’s BMI, the expressions of mir-126 in aorta samples in patients with BMI lower than 30 was 20.3±0.9 and in patients with BMI upper than 30 was 19.6±0.3. This expression level was statistically different in two groups (*P* < 0.05, 95% CI 0.06 to 1.2). The results showed that the expression of mir-145 was also different in two groups (10.6±0.6 versus 11.2±0.5) (*P* < 0.05, 95% CI -1.0 to -0.14). It is showed that the expression of mir-133a, however, was not different in two groups (*P* > 0.05, 95% CI -1.2 to 0.4). The expression of miRNAs in the saphenous vein is also shown in Table 3. It showed that the expression of mir-126 in saphenous vein samples in two patients group was not different (*P* > 0.05, 95% CI -0.23 to 1.5). The expression of mir-133a in two patients group was also not different (*P* > 0.05, 95% CI -0.5 to 0.23). The results showed that the expression of mir-145 in the saphenous vein samples of the patients with BMIs lower than 30 was significantly higher than the patients with BMIs upper than 30 (*P* < 0.05, 95% CI -0.8 to -0.03).

**Table 3:** Expression of miRNAs in aorta and saphenous vein analyzed considering BMIs of the patients

	**BMI<30** **(n=19)**	**BMI>30** **(n=11)**	P value* ^#^
Aorta			
mir-126	20.3±0.9**	19.6±0.3**	<0.05
mir-133a	17.9±1.1**	18.3±0.8**	>0.05
mir-145	10.6±0.6**	11.2±0.5**	<0.05
Saphenous			
mir-126	14.3±1.1**	13.7±1.1**	>0.05
mir-133a	16.1±0.5**	16.3±0.5**	>0.05
mir-145	7.1±0.5**	7.5±0.3**	<0.05

*Values are obtained by independent sample *t* test. ^#^ Exact *P* value of <0.05 with was considered as statistically significant. ** Values are express as Mean± SD

## Discussion

In this study we showed that the mir-126, mir-133a and mir-145 are variably expressed in the aorta and saphenous vein samples from CAD patients. Moreover, we reported that mir-126 is significantly under expressed in aorta samples of overweight patients. Over the past decade several studies have shown abnormal expression pattern of miRNAs in cardiovascular disease such as cardiac hypertrophy, arrhythmias^21^ and MI.^22^ There have also shown that many other miRNAs have roles on development of atherosclerosis and play a crucial role in lipoprotein metabolism.^22,23^

In this study, we found that the expression pattern of mir-126 was different in aorta and saphenous vein samples. Mir-126 was more highly expressed in the saphenous vein. It has shown that mir-126, an endothelial-specific microRNA, regulates many aspects of endothelial cell biology including cell survival and required for governing vascular integrity and maintenance.^24^ Furthermore, recent studies have shown that mir-126 promotes angiogenic signaling pathways.Fish et al showed that mir-126 is highly expressed in endothelial cells and directly targets Sprouty-related, EVH1 domain-containing protein 1 (SPRED1), vascular cell adhesion protein 1 (VCAM1), and phosphoinositide-3-kinase regulatory subunit 2 (PIK3R2) and function to promote vascular endothelial growth factor (VEGF) signaling pathway.^25^ In addition, it has also shown that VEGF plays a critical role in cardiac angiogenesis following MI.^25^ Moreover other genes regulated by mir-126 may contribute in responsiveness of endothelial cells to growth factors^26,27^ and platelet-derived growth factor (PDGF-AB) limits the extent of MI.^27^ Thus the lower expression of mir-126 in endothelial cells of aorta may involve in development or progression of endothelial dysfunction in CAD patients. In addition to these findings, we found that mir-126 expression in overweight patients even is lower. Despite the higher circulating growth factor levels in patients with higher peri-aortic fat,^28^ the lower expression of mir-126 may nullify the effect of the growth factor. While the increase in VEGF level may have an angiogenic effect, this may lead to atherosclerosis and plaque instability, a complication that may relate to lower expression of mir-126 in endothelial cells.^29,30^

Fichtlscherer et al found that circulating mir-133a was significantly increased in CAD patients. They concluded that mir-133 may be of cardiac muscle origin.^31^ mir-133a is believed to be specifically expressed in cardiac and muscle tissue and control myogenesis by regulating differentiation process and myoblast proliferation.^32^ In contrast to these findings, we showed that mir-133a highly expressed in aorta. Mir-133a exerts its critical role in cardiomyocyte proliferation by regulating apoptosis, and it has shown that mir-133a is anti-apoptostic.^33^

Izarra et al showed that mir-133a improves the protective capacity of cardiac progenitor cells (CPCs) in MI and exposure to cardiac differentiation progressively increases the expression of miR-133a. These researchers also found that this effect of mir-133a is mediated by preventing apoptosis through caspase 3 inhibition.^34^ Interestingly, it was shown that the target genes of mir-133a include *Bmf* (*Bcl-2* modifying factor) and *Bim* (*Bcl2l11*), which are potent pro-apoptotic factors.^35^ Remarkably, some miRNAs found to be up-regulated in diseased tissue were down-regulated in the circulation. In current study miR-145 was up-regulated in atria while Fichtlscherer et al showed smooth muscle cell (SMC-derived) miR-145 was down-regulated in the plasma of patients with CAD^31^; However Cipollone et al demonstrated up-regulation of miR-145 in unstable atherosclerotic plaques.^17^ Their finding was in accordance with our data. It is potential that diseased tissue specially uptakes exosomes encompassing those specific miRNAs, so lowering their levels in the blood. Other explanations might contain increased deprivation of circulating miRNAs or their epigenetic silencing.^36^

## Conclusion

Improvement and understanding of the role of miRNAs in cardiovascular physiology and diseases, new miRNA- based therapeutic methods are suggested and established in clinical settings and, optimistically, found effective in the treatment of CAD.

## Acknowledgments

The authors are exceptionally thankful to Tabriz University of Medical Sciences, Tabriz, Iran for financial support and also of all patients who participated in the study. We would also like to thank Dr. Morteza Ghojazadeh for helping us in statical analyses.

## Ethical approval

The study protocol was approved by the ethics committee of the Tabriz University of Medical Sciences (ethical code: IR.TBZMED.REC.1395.1219), and informed consent was taken from each case before enrollment.

## Competing interests

All authors declare no competing financial interests exist.
